# Effects of Resveratrol Supplementation on Delayed Onset Muscle Soreness and Muscle Recovery: A Systematic Review

**DOI:** 10.21315/mjms2024.31.6.7

**Published:** 2024-12-31

**Authors:** Nurdiana Zainol Abidin, Cheong Hwa Ooi, Kazunori Nosaka, Vishanth Rathakrishnan, Siok Yee Chan, Noor Khairiah A Karim

**Affiliations:** 1 Department of Community Health, Advanced Medical and Dental Institute, Universiti Sains Malaysia, Pulau Pinang, Malaysia; 2 School of Medical and Health Sciences, Edith Cowan University, Joondalup, Australia; 3 School of Pharmaceutical Sciences, Universiti Sains Malaysia, Pulau Pinang, Malaysia; 4 Department of Biomedical Imaging, Advanced Medical and Dental Institute, Universiti Sains Malaysia, Pulau Pinang, Malaysia

**Keywords:** resveratrol, trans-3, 5, 4′-trihydroxystilbene, muscle soreness, exercise-induced muscle damage, exercise physiology

## Abstract

Delayed onset muscle soreness (DOMS) and impaired muscle recovery significantly affect athletes and recreational exercisers, influencing their performance and training consistency. Resveratrol, a natural polyphenol known for its anti-inflammatory and antioxidant properties, is thought to mitigate these effects, yet its effectiveness remains to be fully verified. This systematic review evaluates the impact of RES supplementation on muscle recovery in adults by examining its influence on DOMS, oxidative stress, and inflammation, along with its interactions with other supplements. Three electronic databases and one registry were searched in October 2023. A total of 10 studies met the inclusion criteria, encompassing a combined participant count of 238 (N=238). The review encompassed diverse participant populations, exercise types, and resveratrol dosages. The findings indicated that resveratrol potentially reduces markers of muscle damage, such as creatine kinase and lactate dehydrogenase, and alleviates DOMS symptoms to varying degrees. However, results varied based on exercise intensity, participant demographics, timing of supplementations and dosages. Synergistic interaction studies suggested that resveratrol, in combination with other compounds, could be more effective in exerting its effects. Despite promising findings, the research was limited by diverse study designs and the absence of long-term impact assessments. Further studies should standardise methods and explore resveratrol’s long-term safety and effectiveness. Nevertheless, these results underscore resveratrol’s potential as a beneficial supplement in exercise and sports medicine, meriting additional detailed exploration to refine its use.

## Introduction

Delayed onset muscle soreness (DOMS) is a common physiological response experienced not just by athletes, but by anyone who engages in physical activity, especially when the activity is more strenuous or unaccustomed (1). DOMS occurs after unaccustomed exercise due to several factors which include structural damage, disruption of calcium homeostasis, and macrophage activity in the muscle (1). Recent studies suggest that the exact mechanisms behind DOMS and other symptoms of muscle damage are more closely associated with damage to connective tissues, such as fascia, rather than to the muscle fibres themselves, and result from a combination of inflammatory responses and oxidative stress within the affected muscles (2). DOMS typically appears within 24–72 hours post-exercise and often accompany with increased muscle stiffness (3). Other symptoms include a prolonged decrease in muscle function, evidenced by reduced maximum voluntary contraction; noticeable swelling; and a rise in specific blood markers such as creatine kinase (CK) and myoglobin indicating tissue damage (2). While many view DOMS as a temporary inconvenience, its effects can hinder individuals from maintaining regular exercise routines, potentially impacting their health goals and overall well-being.

In recent years, there has been a growing focus on plant-derived molecules (4, 5). This is because they often come with fewer side effects and have multifaceted benefits, making them an area of keen interest for researchers. One such compound that has been in the limelight for its potential health benefits is resveratrol (RES) (4). RES is a naturally occurring polyphenolic compound found in various plants, including grapes, berries, and peanuts. It has gained considerable attention in recent years for its potential health benefits (4, 6). RES has been reported to possess anti-inflammatory, antioxidant, and analgesic effects, which may potentially help alleviate DOMS (6, 7). Some studies suggest that RES may even enhance muscle recovery and adaptation to exercise, further contributing to its potential benefits for exercise-induced muscle damage (6–12). Its potential anti-inflammatory (13) and antioxidant (14) properties make it a promising candidate in the context of challenges faced after engaging in physical activity.

Despite the increasing interest in RES as a potential aid for exercise-induced muscle damage, the current evidence is still in its nascent stages and presents varied findings (7, 15–24). While individual studies provide valuable insights, a systematic review can offer a more comprehensive perspective, identifying consistent findings, highlighting variations, and suggesting areas for future research. This systematic review, therefore, seeks to investigate the overall impact of RES supplementation on exercise-induced DOMS and muscle recovery in adults, exploring how the dosage and timing of supplementation (pre-exercise, post-exercise, or both) affect outcomes. It also examines variations in the effectiveness of RES based on exercise characteristics (type, intensity, duration) and individual differences (age, sex, fitness level), as well as the underlying mechanisms and potential adverse effects associated with its use for mitigating exercise-induced DOMS.

## Methods

Following the guidelines of the Preferred Reporting Items for Systematic Reviews and Meta-Analyses (PRISMA) 2020 (25), a comprehensive and systematic literature search was conducted in Web of Science (WoS), PubMed (Medline), Scopus, and the Cochrane Library (Cochrane Database of Systematic Reviews, Cochrane Central Register of Controlled Trials (CENTRAL)) on 16 October 2023. Methods for conducting this review were pre-specified in a registered protocol on PROSPERO (CRD42023456914) published on 8 September 2023. The search strategy was designed to identify all relevant original articles that investigate the effects of RES on muscle recovery and DOMS indicators post-physical activity or exercise in adults. Appendix 1 details the search strategies performed in the databases. All related articles published from inception up to 16 October 2023 were considered for inclusion. Searches were re-run prior to the final analysis. Unpublished studies were not sought.

**Figure 1:**
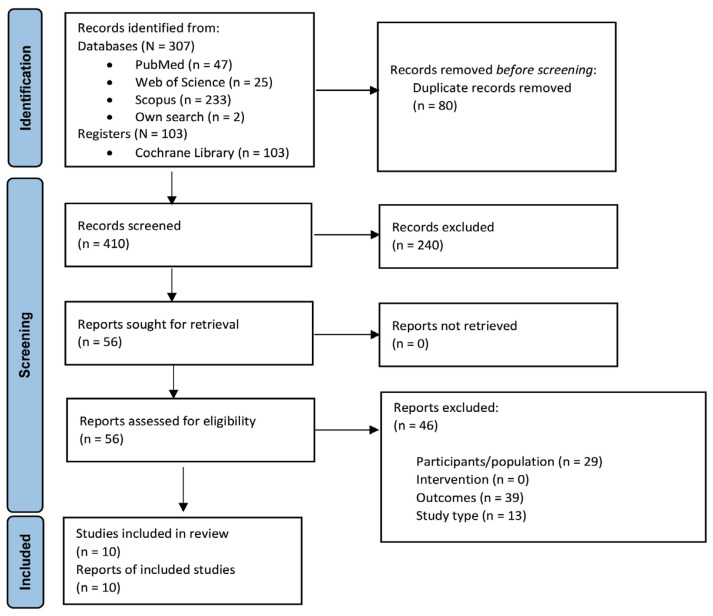
PRISMA flow diagram of search results

Two investigators (NZA and OCH) individually screened the titles and abstracts retrieved from the databases for eligibility. The screening process was performed using Mendeley (version 1.19.4, Mendeley, London, United Kingdom). The number of searches in each database and the number of duplicates were recorded. Duplicates were removed using the built-in function of “check for duplicates” in the Mendeley software. Abstracts that fulfilled the inclusion criteria were retrieved for further screening before obtaining the full-text articles. Thereafter, full articles were reviewed based on inclusion and exclusion criteria. Finally, the two investigators (NZA and OCH) carried out quality assessment and data extraction. In instances where discrepancies arose between the two primary investigators during the review process, resolution was achieved through the intervention of a third investigator (VR). Upon confirming the included studies, their references were also searched manually. The article selection process is presented in Figure 1.

### Inclusion and Exclusion Criteria

Population, Intervention, Control/ comparator, and Outcome (PICO) was used to define the inclusion and exclusion criteria (Table 1). Experimental human trials recruiting adult participants of any gender or ethnicity were eligible for inclusion if they use RES as a dietary or pharmacologic intervention for exercise-induced DOMS. Studies also need to include at least one muscle recovery or muscle damage outcome, range of motion, or muscle performance. Only articles published in full-text English were included. Letters, opinion articles, editorials, reviews, and conference abstracts/ proceedings were excluded. Animal, in vitro, in vivo, and modelling studies were also excluded.

### Assessment of The Characteristics of The Studies

#### Data extraction

Data extraction was performed independently by two authors (NZA and OCH) using a standardised data extraction form. The extracted data include study characteristics (authors, year of publication, country), participant characteristics (age, sex, number of participants, fitness level), intervention details (timing of supplementation, type of RES consumption, dosage, duration, frequency, intensity of exercise), pre- and post-measurements, key findings and quality assessments. These data were extracted and synthesised in Tables 2a and 2b.

#### Quality assessment

The methodological quality of the included studies was evaluated using the Cochrane Risk of Bias Tool 2.0 (RoB 2) (26). Two independent reviewers (OCH and NZA) assessed the risk of bias for each included study using the tool. The RoB 2 is a comprehensive tool for assessing the risk of bias in randomised trials, which evaluates five domains: bias arising from the randomisation process; bias due to deviations from intended interventions; bias due to missing outcome data; bias in the measurement of the outcome; and bias in the selection of the reported result (26). For each domain, the RoB 2 guidelines were followed to answer the signalling questions and make judgements of “low risk”, “some concerns”, or “high risk”. Each question was answered with “yes”, “probably yes”, “probably no”, “no”, or “no information”. The bias risk of each domain was rated as “low”, “some concerns”, or “high.” Similar to the individual domains, overall RoB 2 was also summarised as “low”, “some concerns”, or “high” risk of bias (26). We resolved any disagreements through discussion or by involving a third reviewer (VR). We, then, determined an overall risk of bias for each outcome in each study, following the RoB 2 guidelines (Table 3).

### Participants

The demographic details of participants, including the total number of individuals in each study, their age range, and sex distribution, were documented. Participants were adults who engaged in various exercise regimens, experiencing DOMS.

### Intervention

The intervention of interest was RES supplementation, which could vary in form (e.g., oral capsules, liquid), dosage, and timing relative to exercise (pre-exercise, post-exercise, or both). Studies were included regardless of the exercise regimen followed by participants, provided the RES supplementation was the primary variable being investigated and any co-interventions were uniformly applied across study groups.

### Outcome Measures

Primary outcome measures focused on indicators of muscle recovery and the alleviation of DOMS symptoms. This included standardised scales such as the Visual Analogue Scale (VAS) or the Likert Pain Scale and objective measures of muscle function and recovery. Secondary outcomes considered were recovery markers such as creatine kinase (CK) and lactate dehydrogenase (LDH), oxidative stress markers such as malondialdehyde (MDA), superoxide dismutase (SOD), and glutathione peroxidase (GPx), and inflammatory markers such as C-reactive protein (CRP), interleukin-6 (IL-6), and tumour necrosis factor-alpha (TNF-alpha).

## Results

### Search Results

The database search yielded a total of 410 articles, whereof 10 articles were finally included in this review.

### Characteristics of Included Studies

Table 2a shows the characteristics of the eligible studies that were screened and found suitable for inclusion in the systematic review. The 10 included studies reported data for 238 participants. The age of participants in these studies ranged from 18 to 80 years, providing a broad perspective on the potential effects of RES across different age groups. The studies comprised of more males than females. The studies reviewed were predominantly randomised and controlled, with a mix of double-blind and single-blind methodologies (7, 16–23).

For the intervention methods, RES dosages and the timing of supplementation varied across studies. Pre-exercise supplementation was a common approach (7, 16, 17, 20, 21, 23), ranging from 4 to 90 days prior to exercise, with one study utilising both prior to exercise until post-exercise (24). Conversely, some studies utilised during-exercise 1supplementation (18, 19, 22), ranging from 8-weeks to 3 months of supplementation. This categorisation underscores the diversity in the timing of RES supplementation across various research studies, highlighting the different approaches to understanding its effects in relation to physical activity.

The type and intensity of exercises also varied, which include marathon running (17), high-intensity cycling (16), moderate-intensity ergometer workouts (18), acute plyometric exercises, including maximal vertical jumps (7), moderate-intensity routines incorporating a variety of physical fitness tests (19), resistance training with exercises like leg presses (22), depth jumps and leg presses at high intensity (20) and high-intensity exercises targeting specific muscle groups (21, 23). There was also a study that employed a protocol designed to induce muscle damage through eccentric dorsiflexion (24). This diversity in exercise modalities provides a broad context for understanding the effects of RES supplementation across different physical activities.

Similarly, the outcome measurements are varied across studies. The primary outcomes in the included reviews encompassed measurements of DOMS severity, recovery markers, and inflammatory markers, which include a visual analogue scale (VAS) (17), blood muscle damage biomarkers such as CK and LDH activities (7), and assessments of plasma metabolic response (19) and oxidative stress indicators (20), providing a comprehensive understanding of the physiological impacts of RES supplementation in the context of exercise-induced muscle damage.

### Effects of the Intervention on Study Outcomes

Table 2b highlights the varied effects of RES supplementation on exercise-induced DOMS and related outcomes. The studies reviewed demonstrate a range of responses, from no significant effects (17, 18, 21) to increased muscle strength and resting oxygen consumption (22), decreased subjective fatigue and oxidative damage (22), significant reduction in pain and tenderness and greater energy expenditure (23), reduced pain perception (24), improvements in muscle soreness and exercise performance (7, 20), reduced inflammation (16, 19) and reduced muscle damage indicators (7).

These studies collectively provide insights into the varied effects of RES supplementation on exercise-induced DOMS and related physiological responses. While most studies observed beneficial effects in terms of reduced muscle soreness and inflammation, a limited few found no significant impact, underscoring the complexity of RES’s role in exercise recovery and the need for further research.

### Quality Assessment and Potential Risk of Bias

Table 3 presents a detailed assessment of the risk of bias in the included studies using the RoB 2.0 framework. The analysis reveals that most studies adhered to protocols with minimal deviations, ensuring reliable efficacy assessment. Some studies, however, fail to detail protocol adherence and missing data handling, raising performance and attrition bias concerns. Despite widespread assessor blinding and standardised methods, a few lack measurement transparency, suggesting a need for uniform reporting to accurately gauge RES’s effects and reduce bias.

## Discussion

This systematic review critically examined the role of RES supplementation in modulating DOMS and its overall impact on muscle recovery. Notably, while most studies demonstrate a potential benefit of RES in reducing DOMS severity and enhancing muscle recovery, the variability in outcomes underscores the complexity of the compound interactions within the human body during and after strenuous physical activities. Our review suggests that RES is a promising supplement for improving muscle recovery and mitigating DOMS, with potential benefits for overall performance, although its use may require careful consideration of age, sex, health status, type and intensity of exercise, dosages, and timing of supplementations.

### Efficacy of RES in Reducing DOMS Severity

Theoretical perspectives suggest that RES, known for its anti-inflammatory and antioxidant properties, might mitigate DOMS through reducing muscle inflammation and oxidative stress. This is evidenced from a study by Huang et al. (7), who demonstrated a reduction in muscle soreness in non-athletes engaged in plyometric exercises following RES supplementation. This study highlights the potential role of RES in modulating oxidative stress, which is a key factor in the development of DOMS. Furthermore, the study highlights the importance of RES dosage, revealing that subjects receiving higher doses of RES supplementation reported enhanced recovery metrics, especially following the Wingate anaerobic test. The study’s focus on non-athletes performing high-impact exercises such as plyometrics, which are known to induce significant muscle damage, offers a valuable perspective on the efficacy of RES in a more general population (7). On the other hand, Laupheimer et al. (17) conducted a study investigating the effects of RES supplementation on marathon runners and showed contrasting results. Their findings did not show significant differences in VAS scores for muscle soreness post-marathon nor in their immune responses. The authors stated that this might be due to the low number of participants, the timing of the test, which affected the white blood cell count (WBC) results, and the dose of RES. Regardless, the study is valuable for its hypothesis-driven approach. It suggests that while their study RES dosage may not significantly reduce perceived muscle soreness in marathon runners, it sets a foundation for further research in higher dosages of RES and with higher number of participants.

The nuanced landscape of RES’s impact on DOMS and exercise performance is complex, as evidenced by the varied outcomes in different studies. Two such studies that contribute to this complexity are those conducted by Tsao et al. (16) and Løkken et al. (18), each presenting less conclusive or differing results. Tsao et al. (16) explored the effects of RES supplementation on markers of inflammation, oxidative stress, and fatigue in the context of high-intensity cycling performance. Their findings indicated that while RES supplementation attenuated increases in interleukin-6 (IL-6), a marker of inflammation, after cycling, but it did not significantly impact other blood biomarkers, overall inflammation, oxidative stress, or fatigue. Notably, there was also no significant improvement in high-intensity cycling performance (16). The reduction in IL-6, however, could be advantageous for athletes in training, particularly for endurance performance where inflammation plays a crucial role. This suggests that RES might have specific benefits in modulating certain aspects of the inflammatory response as soon as 4 days of ingestion pre-workout, albeit without ergogenic effects on performance (16).

On the other hand, Løkken et al. (18) investigated the effects of RES on exercise capacity in patients with mitochondrial myopathy (MM). Their findings were that RES did not enhance exercise capacity, maximal oxidative capacity, or workload capacities in these patients. Furthermore, RES failed to increase fatty acid oxidation or improve perceived fatigue as measured by the fatigue severity scale, nor did it improve self-assessed health (18). Additionally, RES did not elevate biomarkers in muscle tissue or increase the activity of mitochondrial complexes I and IV in peripheral blood mononuclear cells (PBMCs). This study is particularly important as it highlights the limitations of RES’s efficacy in a clinical population with specific metabolic impairments, suggesting that its benefits may not extend to all forms of exercise-induced stress or fatigue (18). The limited impact observed in these studies could be attributed to the complex interplay between RES’s bioavailability, the intensity of exercise, and individual physiological differences. This aligns with the concept that the efficacy of nutritional interventions like antioxidants in DOMS management can vary significantly (27).

### Effects of RES on Muscle Recovery and Performance

The effects of RES on muscle recovery can be understood by examining its impact on key biomarkers of muscle damage and the underlying cellular processes. A primary indicator of muscle damage is the level of CK in the blood (15). CK is an enzyme found in the heart, brain, and skeletal muscle, and its elevated levels post-exercise indicate muscle tissue damage. When muscle fibres are damaged, CK is released into the bloodstream, serving as a reliable marker of muscle injury. In addition to CK, LDH is another enzyme that plays a crucial role in energy production and is released into the blood when cellular damage occurs (15). Elevated levels of LDH post-exercise are also indicative of muscle damage. The mechanism of action of RES in this context is multifaceted. As an antioxidant, RES helps in scavenging free radicals produced during intense exercise, thereby reducing oxidative stress in muscle cells (28). Oxidative stress can lead to cellular damage and inflammation, exacerbating muscle soreness and damage. Therefore, by mitigating oxidative stress, RES not only protects muscle cells from further damage but may also limit the release of CK and LDH, promoting muscle recovery and reducing inflammation (29). In terms of inflammation, RES is known to modulate various inflammatory pathways. Muscle damage induced by exercise triggers an inflammatory response, which is a natural part of the healing process but can also contribute to pain and prolonged recovery times. RES’s anti-inflammatory properties, thereby, could potentially reduce the severity of the inflammatory response and aiding in quicker muscle recovery (13).

In the study by Huang et al. (7), the supplementation with RES was found to significantly reduce and accelerate the recovery of muscle damage indicators such as CK and LDH activities in the blood after exercise-induced muscle damage (EIMD). This suggests that RES may facilitate quicker recovery by attenuating cellular damage through its antioxidative and anti-inflammatory actions. The reduction in CK and LDH levels indicates that RES may help in preserving the integrity of muscle cell membranes, reducing the leakage of these enzymes into the bloodstream. This protective effect on muscle cells can be crucial for athletes and individuals engaged in regular or intense physical activity, as it may lead to reduced soreness and faster recovery times. Similarly, Jo et al. (20) noted a trend of reduced localised hyperalgesia and DOMS, with a slight efficacy in performance preservation during EIMD, though they observed no significant attenuation of CK leakage.

However, the impact of RES on performance metrics is more nuanced. The study by Tsao et al. (16) reported that while RES supplementation attenuated exercise-induced increase in IL-6, it did not significantly improve high-intensity cycling performance. Likewise, Løkken et al. (18) observed that RES did not enhance exercise capacity or maximal oxidative workload capacities in patients with MM. These findings suggest that while RES may assist in the biochemical recovery process, its direct influence on performance metrics like endurance and strength may not be as pronounced.

### Attributed Factors to the Variability in Responses and Study Outcomes

The effects of RES on DOMS, muscle recovery and performance highlight a notable variability in study outcomes, which can be attributed to several factors. These include differences in RES dosages, diverse participant characteristics, and the varied nature and intensity of exercises involved in the studies. Understanding these variations is crucial for interpreting the efficacy of RES in different contexts.

One of the primary factors contributing to the variability in study outcomes is the dosage of RES used. The relationship between RES dosage and its effectiveness is complex and appears to be dose-dependent, as suggested by studies such as Huang et al. (7), which observed more pronounced recovery effects with higher doses. In contrast, lower doses in studies like Macedo et al. (19) may not have been sufficient to elicit significant changes, indicating that the dosage is a critical factor in determining the efficacy of RES supplementation.

The age, sex, health status and fitness level of study participants also influence the outcomes of RES supplementation. Different age groups, sexes and health statuses may respond differently to RES due to variations in metabolism and baseline physiological states. For example, the response in older adults with MM in Løkken et al. (18) contrasted with the younger, non-athletic group in Huang et al. (7). There are a few hypothetical reasons for this. Research has shown that RES’s effects may be muscle fibre type-specific (10). Evidence from animal studies suggests that RES affects differently on type IIA and IIB fibres, with larger fibres observed in the recovery group. Since MM often affect specific fibre types differently, RES’s selective influence on muscle fibre types could lead to varied outcomes in muscle recovery and function in affected individuals (10). Furthermore, in older adults with underlying mitochondrial disorder (18), the metabolic rate is likely slower compared to younger, healthy individuals. This can affect how RES is metabolised and utilised in the body. Additionally, ageing is associated with increased oxidative stress and a chronic low-grade inflammatory state known as “inflammaging” (30). The effectiveness of RES in such a context may be limited or require higher dosages to overcome this baseline inflammatory state and oxidative stress.

RES may also affect differently on people with different fitness levels, which can be seen in the contrasting results by Laupheimer et al.(17) and Huang et al. (7). In this case, trained athletes (17) might exhibit different baseline levels of oxidative stress and inflammation compared to untrained individuals (7), influencing their response to supplementation (31). Non-athletes, typically characterised by lower baseline fitness levels, may respond differently to RES supplementation compared to endurance athletes, who usually have higher fitness levels and are accustomed to regular, intense physical activity. The study by Huang et al. (7) found that RES had a noticeable effect on reducing muscle soreness and improving recovery in nonathletes. This group may benefit more distinctly from RES’s anti-inflammatory and antioxidant properties due to their bodies’ potentially greater susceptibility to exercise-induced oxidative stress and inflammation, given their lower baseline oxidative stress tolerance. Conversely, the research by Laupheimer et al. (17) involving endurance athletes—individuals who regularly engage in high-intensity, prolonged physical activity—may not have shown as pronounced benefits from RES supplementation. Endurance athletes often develop adaptive mechanisms to manage oxidative stress and inflammation more efficiently due to their training, possibly diminishing the relative impact of RES’s antioxidative and anti-inflammatory effects. The contrasting results between these two studies underscore the importance of considering individual fitness levels when evaluating the efficacy of RES. This insight suggests that RES supplementation may need to be tailored to the individual’s fitness background to maximise its potential benefits.

In addition to age, health status and fitness level, sex differences have also been shown to play a role in the response to RES (32, 33). Hormonal variations, particularly the effects of oestrogen, has been found to influence antioxidant capacity and inflammatory responses. Women, for instance, might respond differently to RES supplementation due to the protective cardiovascular and antioxidative effects of oestrogen, which could interact with RES’s mechanism of action (32, 33).

Finally, one might also consider the type and intensity of exercise as a factor in variability of responses to RES. Different exercises induce varying levels of muscle damage, oxidative stress, and inflammation, which in turn affect the body’s response to RES. The high-intensity cycling challenge in Tsao et al. (16) might induce different physiological responses compared to the plyometric exercises in Huang et al. (7), suggesting that the nature of physical exertion is an important consideration in evaluating RES’s effectiveness. Individual biological differences, including genetic predispositions and lifestyle factors, can also contribute to the diverse responses seen in RES studies (34). These individual factors can affect how RES is metabolised and the body’s overall response to its supplementation, adding another layer of complexity to the interpretation of study outcomes.

### Potential Mechanisms Underlying the Role of RES in the Modulation of Oxidative Stress, Inflammation, and Cellular Functions

**Figure 2:**
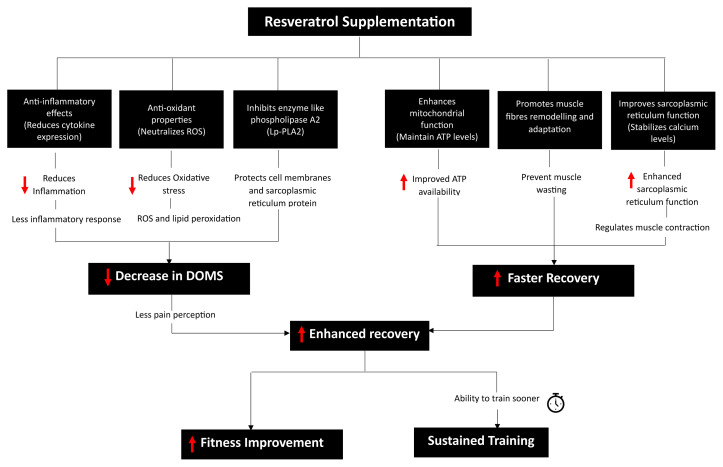
Mechanisms of RES in alleviating DOMS and enhancing muscle recovery

The potential mechanism underlying the role of RES in modulating oxidative stress, inflammation, and cellular functions particularly in the context of exercise recovery, is rooted in its ability to interact with and influence various cellular pathways (Figure 2) (6, 35). RES’s multifaceted role in muscle recovery extends from modulating enzyme activity to enhancing cellular functions. For example, it has been found to potentially inhibits enzymes such as phospholipase A2 (36), thus safeguarding cell membranes and sarcoplasmic reticulum proteins from damage. Additionally, its ability to bolster mitochondrial function (29) may preserve ATP levels, which are vital in preventing muscle damage. Beyond energy dynamics, RES also encourages muscle fibre remodelling and adaptation (37), possibly by influencing growth factors while simultaneously reducing cell death. It also plays a crucial role in muscle function by contributing to calcium homeostasis (38) within the sarcoplasmic reticulum, a key factor in muscle contraction and a determinant in the development of muscle soreness. At the cellular level, RES is also known to activate pathways associated with cellular protection and repair. One key mechanism is its ability to upregulate the expression of antioxidant enzymes. These enzymes, such as superoxide dismutase (SOD) and catalase, play a critical role in neutralising reactive oxygen species (ROS) generated during intense physical activity (39, 40). By enhancing the body’s natural antioxidant defences, RES can help mitigate the oxidative damage to muscle cells that often occurs during and after high-intensity exercise.

In addition to its antioxidative properties, RES has also been found to downregulate NF-κB (41), inhibit the IGF-1R/Akt/Wnt signalling pathway, and stimulate the expression of p53 (42), all of which contribute to its anti-inflammatory effects (43). Previous studies have also demonstrated that RES supplementation can alleviate the detrimental effects associated with EIMD during the recovery phase (6). In an animal study, Wu et al. (15) observed that RES supplementation effectively enhanced muscle strength and endurance performance while also reducing biochemical markers of fatigue following exercise. Moreover, RES has also been found to exert a synergistic effect with resistance exercise training, whereby the combination was found to expedite the recovery of muscle strength and significantly boost muscle endurance performance (44). Additionally, RES supplementation post-muscle contusion has been found to be effective in decreasing damage indicators such as increased CK and LDH activities in the blood and in promoting the regeneration of muscle satellite cells (12). However, these studies were conducted using animal models, and further research is necessary to confirm these benefits for human muscle recovery.

The potential mechanism underlying RES’s role in exercise recovery involves its ability to enhance the body’s antioxidative defences and reduce inflammation. This dual action is crucial in mitigating the detrimental effects of EIMD.

### Synergistic Effects of RES with Other Nutritional Supplements

The consumption of multiple supplements simultaneously is a common practice among athletes. Recent research suggests that when combined with other nutritional supplements or interventions, RES may exhibit synergistic effects, enhancing its efficacy in various health contexts (45–47). To date, no study has provided evidence of the synergistic effects of RES with other nutritional supplements in the context of DOMS. However, other context has shown promising results. One area where RES has been found to show promising synergistic effects is in cardiovascular health. Studies have indicated that RES when combined with other red wine phenolics like quercetin, can suppress tissue factor induction in blood cells, which is a key factor in thrombosis and heart attack (48). This suggests that RES, in combination with other compounds, could be more effective in improving cardiovascular health and preventing coronary heart diseases.

In the realm of metabolic health, RES’s combination with other phytochemicals like genistein and quercetin has been shown to synergistically decrease adipogenesis, the process of cell differentiation into fat cells (49). This combination has implications for managing obesity and related metabolic disorders. Additionally, in animal models, the combination of RES with a high-fat diet (50) has demonstrated enhanced metabolic and physiological adaptations, suggesting a potential role in managing obesity and improving exercise performance. RES’s synergistic effects also extend to the field of ageing and neurodegenerative diseases. For instance, its combination with vitamin D has been found to reduced tunicamycin- induced cytotoxicity, reduced elevated endoplasmic reticulum stress, restored insulin signalling disruption, and reduced tunicamycin- induced tau phosphorylation (51). This suggests that RES, in combination with other dietary interventions, could be beneficial in slowing down ageing processes and mitigating age-related diseases.

The synergistic effects of RES with other nutritional supplements or interventions offer promising avenues for enhancing its beneficial impacts on human physiological and metabolic health. These findings underscore the potential of RES as a component of combined therapeutic strategies, although further research is needed to fully understand these interactions and their implications for human health.

### Safety and Adverse Effects of RES Supplementation

In evaluating the suitability of RES supplementation for various populations, addressing safety concerns and potential adverse effects is crucial. The studies reviewed generally indicate that RES is well-tolerated (52), with few significant adverse effects reported (53). However, the long-term safety profile, especially at higher doses, remains insufficiently explored. Notably, studies with diverse dosages and participant demographics did not consistently report detailed information on adverse effects, suggesting a gap in the current literature. To ensure comprehensive understanding and safe application, future research should prioritise detailed monitoring and reporting of any adverse effects, particularly in long-term studies. This focus is essential to ascertain the safety of RES supplementation across different populations, including athletes, individuals with varying health statuses, and those engaged in diverse types of physical activity.

This systematic review comprehensively examines the effects of RES on exercise-induced DOMS and muscle recovery in adults, providing insights into its potential benefits, mechanisms of action, and synergistic interactions with other nutritional supplements.

## Conclusion

In conclusion, while RES emerges as a potential supplement for enhancing muscle recovery and mitigating DOMS, its application must be approached with a nuanced understanding of its variable efficacy and the current limitations in research.

### What Was Already Known on This Topic

DOMS poses significant recovery challenges for athletes and recreational exercisers, impacting subsequent performance and training continuity. Previous studies have explored various nutritional and pharmacological interventions to mitigate DOMS, with RES, known for its anti-inflammatory and antioxidant properties, showing potential benefits. However, these studies have often presented mixed results, highlighting the need for a more comprehensive analysis that includes a wider range of studies.

### What This Review Adds

This systematic review provides a thorough examination of RES supplementation’s effects on exercise-induced DOMS and muscle recovery. Our findings indicate that RES can potentially reduce DOMS severity and enhance muscle recovery, primarily through its antioxidant and anti-inflammatory actions. The review also identifies significant variability in the effectiveness of RES, influenced by factors such as age, sex, health status, fitness level, dosage, timing of supplementation, and the type of exercise. Importantly, this review highlights the potential for synergistic effects when RES is combined with other nutritional supplements, suggesting a promising area for future research. By offering a detailed analysis of the current evidence, this review clarifies the conditions under which RES supplementation could be most beneficial for athletes and recreational exercisers, contributing to the optimisation of recovery strategies in sports and exercise medicine.

## Figures and Tables

**Table 1:** Eligibility criteria for identified studies according to PICO

Properties	Eligibility criteria
Type of articles	Original research articles
Language	English
Population	Adults (18 years and above) who engage in exercise or physical activity that can induce DOMS
Intervention	Consumption of RES
Control/comparator	No RES consumption or placebo
Outcomes	Primary Outcomes: DOMS severity: Changes in DOMS severity will be the primary outcome. This can be evaluated using standardised scales such as the Visual Analogue Scale (VAS) or the Likert Pain Scale. Secondary Outcomes: Recovery markers: Changes in recovery markers such as creatine kinase (CK) and lactate dehydrogenase (LDH) levels. Inflammatory markers: Changes in inflammatory markers such as C-reactive protein (CRP), interleukin-6 (IL-6), and tumour necrosis factor-alpha (TNF-alpha). Oxidative stress markers: Changes in oxidative stress markers such as malondialdehyde (MDA), superoxide dismutase (SOD), and glutathione peroxidase (GPx).

**Table 2a:** Characteristics of the eligible studies (author, year, country, participants’ number, age range, gender, fitness level, study design, intervention timing of supplementation, type, intensity of exercise, pre- and post-measurements)

No	Author(s)/ year/country	Participants (sample size: n = completed/ enrolled)/age range/ gender/fitness level)	Study design	Intervention (timing of supplementation/ type/ intensity of exercise)	Pre- and post-measurements
1	Laupheimer et al. 2014) England (17)	7/8 (viral infection)/ 41–55 years/male/ athletes	Randomised, double-blind, placebo-controlled pilot study	Participants were randomly assigned to take either 600 mg of RES (n = 3) or a placebo (n = 4) daily for 7 days before a marathon, with the RES group consuming two 100 mg tablets three times a day, ending on the race day. They were advised not to change their diet and received no nutrition or hydration guidance before the race.	Pre- and post-marathon measurements included blood samples for white blood cell count (WBC) and C-reactive protein (CRP), and a visual analogue scale (VAS) for DOMS. These measurements were taken 48 hours before and 18–32 hours after the marathon.
2	Huang et al. (2021) Taiwan (7)	36/36/21.09 ± 1.33 years/male/non-athletic	Double-blind, placebo-controlled trial	The study divided participants into three groups: one received a placebo (n = 12, 1000 mg/day of methylcellulose), the second 500 mg/day of RES (n = 12) and 500 mg/day of methylcellulose (RES-500), and the third 1000 mg/day of RES (RES-1000, n = 12). They took their supplements twice daily for 10 days post-baseline assessment. This was followed by an acute plyometric exercise session (10 sets of 10 maximal vertical jumps, interspersed with a one-minute recovery between each set) to induce muscle damage, after which performance, muscle soreness, and damage biomarkers were measured for 72 hours.	Anthropometry, body composition, exercise performance (CMJ, WAnT), muscle soreness (PPT), muscle damage biomarkers (CK, LDH), clinical biochemistry (AST, ALT, BUN, CREA, CBC).
3	Tsao et al. (2021) Taiwan (16)	8/8/19.2 ± 0.5 years/ male/physically active	Single-blind, randomised, controlled, crossover study with a 7-day washout period between each trial	Participants were randomly assigned to take either 480 mg of RES daily for four days (totalling 1920 mg) or a placebo. This was followed by a high-intensity cycling challenge, conducted 60 minutes after the final RES dose, where they cycled at 80% of their maximal oxygen consumption (VO2max) at 60 rpm.	Blood samples for glucose, NEFA, LDH, CK, UA, TAC, MDA, TNF-α, IL-6; Maximal oxygen consumption test. Blood samples were collected before, during, and after the exercise.
4	Løkken et al., (2021) Denmark (18)	10/11 (unrelated illness)/18–80 years/ mixed gender adults/ ambulatory patients with genetically verified MM	Randomized, double-blind, crossover, placebo-controlled	Participants were split into two groups: one received 1000 mg of RES first (n = 6) daily in two 500 mg doses at breakfast and dinner for 8 weeks; the other received a placebo first (n =5). This was followed by a 4-week washout period, then the groups switched treatments for another 8 weeks. During this period, all participants exercised for 20 minutes on a cycle ergometer at 60% of their maximal oxygen consumption, gradually increasing to maximal workload within 4–8 minutes.	HR, VO2max, lactate concentrations, SF-36, FSS, perceived exertion (Borg scale), activities of electron transport chain complexes I and IV in PBMC, GDF-15, FFA and Pyruvate, mitochondrial biomarkers in muscle tissue (SIRT1, AMP-activated protein kinase [AMPK], PCG1α), creatine, GFR, potassium, sodium, creatine kinase, myoglobin, ALT, AST, bilirubin, and amylase.
5	Macedo et al. (2015) Brazil (19)	60/60/21–22 years/ male/military firefighters	Placebo-controlled, double-blind	Participants were assigned to two groups: one received maltodextrin capsules (placebo, n = 30) and the other 100 mg/ day of RES (n = 30). The participants took one capsule every morning for 90 days, alongside their regular 2-hour daily moderate-intensity exercise routine. The study included a physical fitness test (chin-ups, sit-ups, 50-metre sprint, and a 12-minute run) conducted before and after the supplementation period.	Plasma metabolic response (Glucose levels, TG, Total cholesterol, LDL, HDL, CK, LDH, Serum iron concentration, AST, ALT, GGT, UA), oxidative stress indicators (Thiol groups, 8-isoprostane, 8OHdG, FRAP assay, Erythrocyte enzyme activities), inflammatory cytokines (IL-6, TNF-α, IL-1β).
6	Jo et al. (2019) USA (20)		Randomised, double-blind, placebo-controlled, between-group	Participants were randomly divided into a RES group (n = 10) and a control group (CTL) (n = 12). The RES group received a daily 716 mg polyphenol blend capsule, including 60 mg of trans-RES, sourced from various extracts like muscadine grape, red wine, and pomegranate. The CTL group received a placebo capsule matching in taste and appearance. After 30 days, both groups performed an exercise routine to induce muscle damage, consisting of five sets of 20 vertical depth jumps and two sets of six leg press repetitions at 90% of their one-repetition maximum (1RM).	Body composition (TBM, LM, FM, BF%, BMI), muscle soreness (VAS both at rest and under muscular tension), pain threshold and tolerance, range of motion, performance (stand-and reach, mean power (MP) and peak power (PP) during a leg press exercise), serum biomarkers (CK, CRP, IL6). Measured before the supplementation period, immediately after the 30-day supplementation, and at 24- and 48-hours post-exercise.
7	O’Connor et al. (2013) USA (21)	40/40/18–35 years/ mixed genders/ recreationally active	Randomised, double-blind, placebo-controlled trial	Participants were randomly split into two groups: one received a daily drink from freeze-dried grape powder (46 g) containing phytochemicals like RES and anthocyanins (n = 20), and the other a caloric equivalent placebo (n = 20) without polyphenols. They consumed these drinks for 45 days. The grape powder was made from a mix of red, green, and blue-black California grapes. After 42 days, they performed 18 high-intensity exercises of the nondominant elbow flexors. Measurements were taken before, after the supplementation period, and following the exercise.	VO_2_max, work capacity (treadmill performance time), mood (Profile of Mood States questionnaire), SF-36 Health Survey, inflammation (arm volume changes), pain (VAS), arm muscle function (Isometric Strength of the Elbow Flexors, ROM), BC, 7-day physical activity recall questionnaire, Harvard Food Frequency Questionnaire.
8	Kawamura et al. (2021) Japan (22)	26/26/22.3 ± 0.3 years/ male/not habituated to regular exercise	Randomised controlled trial	Participants were split into a control group (n = 13) and an intervention (n = 13) group, the latter receiving dietary supplements including salmon flakes, green and yellow vegetable juice, and lingonberry jam (rich in RES) for 10 weeks. Both groups followed a twice-weekly resistance training programme for the same duration, consisting of eight exercises (chest press, fly, leg press, extension, curl, back extension, rowing, sit-up) on a Senoh combined machine. Each session involved three sets of ten reps at 10 repetition maximum (RM), with 2–3 days intervals between sessions and progressively increasing loads.	Body composition (BMI, %BF, SMM) nutrient intake, maximal voluntary contraction (MVC), subjective fatigue (VAS), oxygen consumption, serum carbonylated protein level.
9	Udani, Singh, Sandoval (2008) USA (23)	10/10/18–45 years/ mixed gender/untrained subjects	Randomised, double-blind, placebo-controlled, crossover pilot study	The participants were divided into two groups in a crossover design: one receiving the Bounce-Back™ supplement (contains RES, n = 5) and the other receiving a placebo (n = 5). The supplementation was administered for 33 days, with a specific focus on the days leading up to and following a standardised eccentric exercise protocol. This protocol consisted of isokinetic quadriceps squat contractions performed on day 30. After a two-week washout period, the groups were crossed over to the other intervention arm.	Pain (VAS) and tenderness evaluations (algometer), blood draws (pre-exercise, immediately post-exercise, 6, 24, 48, 72 hours post-exercise), TEE, MAEE, serological markers of muscle damage (CPK and Myoglobin).
10	Kristoffersen, Christensen, Oliveira (2022) Denmark (24)	18/18 (male: n = 10, female: n = 8)/~23 years/untrained	Randomised controlled trial, blinded	Participants were randomly assigned to either the intervention group (n = 9) or the placebo group (n = 9). The intervention group received a dosage of 500 mg/ day of RES for three days prior to the exercise test. In contrast, the placebo group was given 400 mg/day of calcium capsules, following the same schedule. The RES or placebo supplementation continued from 3 days prior to the first test until the post-test, spanning a total of five days. The exercise regimen consisted of a 10 × 10 maximal eccentric dorsiflexion protocol designed to induce muscle damage and subsequent DOMS.	PPT, VAS, isometric ankle dorsiflexion torque, EMG mean power frequency; assessed before (PreEIMD) and 24 hours after the exercise (PostEIMD) to assess the impact of RES supplementation on pain perception and muscle function post-exercise.

Note: CMJ = countermovement jump; WAnT = wingate anaerobic test; PPT = pressure pain threshold; CK = creatine kinase; LDH = lactate dehydrogenase; AST = aspartate aminotransferase; ALT = alanine aminotransferase; BUN = blood urea nitrogen; CREA = creatine; CBC = complete blood count; NEFA = non-esterified fatty acids; UA = uric acid; TAC = total antioxidant capacity; MDA = malondialdehyde; HR = heart rate; SIRT-1 = sirtuin 1; GFR = glomerular filtration rate; TNF = tumour necrosis factor; IL = interleukin; MM = mitochondrial myopathies

Note: TG = triglycerides; LDL = low-density lipoprotein; HDL = high-density lipoprotein; CK = creatine kinase; LDH = lactate dehydrogenase; GGT = gamma-glutamyl transferase; AST = aspartate aminotransferase; ALT = alanine aminotransferase; UA = uric acid; FRAP = Ferric Reducing Antioxidant Power; TNF = tumour necrosis factor; IL = interleukin; TBM = total body mass; LM = lean mass; FM = fat mass; BF = body fat; BMI = body mass index, VAS = visual analogue scale; CRP = C-reactive protein; ROM = rang of motion; BC = body composition

Note: BMI = body mass index; BF = body fat; SMM = skeletal muscle mass; VAS = visual analogue scale; TEE = total energy expenditure; MAEE = measured active energy expenditure; CPK = creatine phosphokinase; PPT = pressure pain threshold; EMG = electromyography; EIMD = exercise-induced muscle damage

**Table 2b:** Characteristics of the eligible studies (author, year, country, duration of study, baseline characteristics, dosage of resveratrol, key findings)

No	Author(s)/ year/country	Duration of study	Baseline characteristics	Dosage of resveratrol	Key findings
1	Laupheimer et al. (2014) England (17)	7 days immediately before the marathon	Male athletes, similar training load and weight, no chronic inflammatory medical conditions, muscle disorders, heart conditions, or history of immune suppressants or anti-inflammatories.	600 mg daily for 7 days	No significant differences in immune response or DOMS between RES and placebo groups after the marathon. Further investigations suggested with longer treatment time and higher doses, including additional parameters such as interleukins.
2	Huang et al. (2021) Taiwan (7)	10 days + 72 hours post-exercise for tracking	Medical histories, exercise habits were recorded, and anthropometry, exercise performance, and muscle soreness were measured 10 days before placebo or RES supple mentation.	Trans-RES (Biotivia Inc., New York, NY, USA), 500 mg/day and 1000 mg/ day	At 72 h post-EIMD, the FP and RFD of the CMJ in RES groups showed no significant difference compared to that at baseline but was significantly greater than the placebo group. In the WAnT, RES group had a better recovery effect on the RPP, RMP and FI, especially in the high-dose group. For the detection of muscle pain after PEIMD, the RES group was significantly better than the placebo group. In addition, for muscle damage indexes, such as CK and LDH, after PEIMD, supplementation with RES could significantly reduce and accelerate recovery. Results suggest that replenishing RES in advance could effectively reduce muscle pain, increase exercise performance, and decrease muscle damage indicators caused by PEIMD, and the recovery was faster.
3	Tsao et al. (2021) Taiwan (16)	4 days	Physically active students, average VO_2_max 51.4 ± 1.7 mL/kg/min.	RES capsule 480 mg/day (total 1920 mg) (Taiwan Jellyfig Biotechnology Corporation, Kaohsiung, Taiwan)	RES supplementation attenuated exercise-induced IL-6 but not blood biomarkers, inflammation, oxidative stress or fatigue. No significant improvement in high-intensity cycling performance. Inflammation plays a crucial role in endurance performance, so this discovery might be advantageous for athletes in training for major competitions.
4	Løkken et al. (2021) Denmark (18)	20 weeks (8 weeks intervention, 4 weeks washout, 8 weeks opposite treatment)	Medical history (including muscle biopsy readings from time of diagnosis and previous cardiac assessments), a neurological examination, vital sign measurements and an ECG, varying degrees of exercise intolerance and fatigue.	1000 mg/day (trans-RES, purity >98%, Evolva^™^).	RES does not enhance exercise capacity in patients with MM, nor does it boost maximal oxidative or workload capacities. Additionally, RES fails to increase FAO in these patients. From a patient perspective, RES does not improve perceived fatigue (FSS), or self-assessed health. RSV does not elevate biomarkers in muscle tissue, nor does it increase the activity of mitochondrial complexes I and IV in PBMC.
5	Macedo et al. (2015) Brazil (19)	90 days	Male military firefighters, moderate intensity regular exercise, dietary intake.	100 mg/day (98% trans-RES derived from Polygonum cuspidatum by Farmel Pharmacy, Brazil).	Resveratrol showed an anti-inflammatory effect, reducing IL-6 and TNF-a levels. No significant effects on oxidative stress biomarkers or antioxidant defence systems.
6	Jo et al. (2019) USA (20)	30 days	Age: 23.4 ± 2.7 years (CTL), 22.3 ± 3.0 years (RES); resistance-trained.	60 mg/serving of trans-RES in a 716 mg/serving polyphenol blend.	Slight efficacy in performance preservation during EIMD, no significant attenuation of CK leakage, localised hyperalgesia and DOMS lessened, potential anti-inflammatory effects.
7	O’Connor et al. (2013) USA (21)	45 consecutive days	Excluded pregnant women, individuals engaged in vigorous activities regularly, those with contraindications to exercise, those taking pain or prescription drugs (except oral contraceptives), and high consumers of polyphenol-rich products.	Grape powder used, not isolated resveratrol. 1.75 mg/kg of RES per kilogram of grape powder (provided by the California Table Grape Commission).	No significant effect of grape consumption on VO_2_max, work capacity, mood, perceived health status, inflammation, pain, or physical function responses to mild eccentric-exercise-induced muscle injury.
8	Kawamura et al. (2021) Japan (22)	10 weeks	Healthy, non-smoking, no chronic disease or medication/supplement use	Not specified, but included in the provided foods (lingonberry jam)	Increased muscle strength and resting oxygen consumption in the intervention group. Decreased subjective fatigue and oxidative damage post-training in the intervention group.
9	Udani, Singh, Sandoval (2008) USA (23)	33 days, with a two-week washout period	Healthy, untrained individuals	Not specified (part of Bounce-Back™)	Significant reduction in pain and tenderness post-exercise in the active group. Greater total and active energy expenditure in the active group. Lower, but not statistically significant, serological markers of muscle damage in the active group.
10	Kristoffersen, Christensen, Oliveira (2022) Denmark (24)	1 week (including 3 days of supplementation prior to exercise)	Young, healthy, untrained individuals	500 mg/day	RES reduced pain perception 24 h after exercise, but did not prevent force loss or affect muscle recruitment.

Note: RES = resveratrol; EIMD = exercise-induced muscle damage; FP = force peak; RFD = rate of force development; CMJ = countermovement jump; WAnT = wingate anaerobic test; RPP = relative peak power; CK = creatine kinase LDH = lactate dehydrogenase; IL = interleukin; DOMS = delayed onset muscle soreness; PEIMD = plyometric-exercise-induced muscle damage; RMP = relative mean power; FI = fatigue index

Note: RES = resveratrol; MM = mitochondrial myopathy; FAO = fatty acid oxidation rates; FSS = fatigue severity scale; PBMC = peripheral blood mononuclear cells; EIMD = exercise-induced muscle damage; CK = creatine kinase; IL = interleukin; TNF = tumour necrosis factor; CTL = control; DOMS = delayed onset muscle soreness; ECG = electrocardiogram

**Table 3:** Analysis of risk of bias according to Cochrane Risk of Bias Tool 2.0 (RoB 2) guidelines

References	Bias arising from the randomisation process	Bias due to deviations from intended interventions	Bias due to missing outcome data	Bias in measurement of the outcome	Bias in selection of the reported result	Overall risk of bias
Laupheimer et al. (2014) England (17)	**+**	**+**	**+**	+	+	+
Comments	Random allocation and concealed allocation were performed	No clear deviations reported	More than 85% of initial participants included in analysis	Blinding of assessors was ensured	The study appears to report all expected outcomes	The small sample size and potential for type II error, as mentioned in the study, should be considered for the overall evaluation of the study’s findings.
Huang et al. (2021) Taiwan (7)	+	?	?	+	+	+
Comments	Participants were counterbalanced by performance and divided into groups.	No specific details on adherence or deviations provided.	No information on missing data or how it was handled.	Measurements included various performance and biochemical markers. Double-blind design suggests limited risk of bias in outcome measurement.	The study appears to report all expected outcomes	Based on available information, the study appears to have a structured approach, and low overall risk of bias.
Tsao et al. (2021) Taiwan (16)	?	?	?	+	+	?
Comments	The study design (single-blind crossover) is mentioned, but specific details on the randomisation process are not provided.	Adherence to the resveratrol supplementation and exercise protocol is not explicitly detailed.	Information on dropout rates or missing data during the study is not provided.	The outcomes were measured using standard biochemical assessments, which are likely to be reliable.	The study appears to report all expected outcomes	There are several areas where the risk of bias cannot be determined due to insufficient information.
Løkken et al., (2021) Denmark (18)	+	+	+	+	+	+
Comments	The study was randomised and double-blind, indicating a robust approach to randomisation.	High compliance with the intervention (97% drug compliance reported).	10 out of 11 participants completed the study, suggesting minimal missing data.	Outcomes were measured using standard, reliable methods (e.g., VO_2_max, HR during exercise). Double-blind design suggests limited risk of bias in outcome measurement.	The study appears to report all expected outcomes, including primary and secondary measures.	The study’s design and reporting indicate a rigorous approach to research methodology, enhancing the reliability of its findings.
Macedo et al. (2015) Brazil (19)	?	?	?	?	+	?
Comments	The study was placebo-controlled and double-blind, but specific randomisation methods were not detailed.	Participants were instructed to take the supplement daily, but adherence specifics (e.g., monitoring methods) were not detailed.	No specific mention of handling missing data or dropout rates.	Outcomes were measured using biochemical and physiological methods, but the reliability of these methods in this context is not fully detailed.	The study appears to report all expected outcomes.	The study appears to have a structured design and methodology, but the lack of detailed information on randomisation, adherence monitoring, and handling of missing data raises some concerns regarding the risk of bias.
Jo et al. (2019) USA (20)	?	?	?	?	+	?
Comments	The study mentions random allocation to the RES or CTL group. However, details on allocation concealment and the process of randomisation are not specified.	The study does not provide detailed information on adherence to the supplementation protocol or if any deviations occurred.	The study does not explicitly mention how missing data were addressed, though it notes two participants were dismissed from the study.	Outcomes were measured using standardised methods (e.g., VAS, algometer). However, it’s unclear if outcome assessors were blinded to group allocation.	The study appears to report on pre-specified outcomes related to muscle soreness, flexibility, power, and biomarkers. No evidence of selective reporting is apparent.	While the study appears methodologically sound in several aspects, there are areas where the risk of bias cannot be fully determined due to a lack of detailed information
O’Connor et al. (2013) USA (21)	+	+	+	+	+	+
Comments	Participants were randomly assigned to either the grape or placebo group, with a 1:1 allocation ratio.	High compliance with beverage consumption (99.3% for grape drink and 99.9% for placebo drink).	The study reported no missing outcome data; all 40 participants completed the study and were included in the analysis.	Outcomes were measured using standardised and reliable methods (e.g., VO_2_max test, Profile of Mood States, SF-36 Health Survey).	The study comprehensively reported on all the measured outcomes, with no indication of selective reporting.	The study’s methodology and reporting seem robust, suggesting that the findings are reliable
Kawamura et al. (2021) Japan (22)	+	?	?	?	+	?
Comments	Participants were randomly divided into control and intervention groups. Baseline characteristics such as age and health status were similar.	The study involved regular intake of specific foods and adherence to a resistance training programme. Compliance specifics are not detailed.	The study does not explicitly mention how missing data, if any, was handled.	Outcomes were objectively measured (e.g., muscle mass, MVC, oxygen consumption). However, it’s unclear if outcome assessors were blinded to group allocation.	The study comprehensively reported on all the measured outcomes, with no indication of selective reporting	While the study appears to be well-structured and reports relevant outcomes, the lack of detailed information in certain areas introduces some concerns regarding the risk of bias
Udani, Singh, Sandoval (2008) USA (23)	+	?	?	?	+	?
Comments	Randomised, double-blind, placebo-controlled, crossover study	The study does not provide detailed information on adherence to the supplementation protocol or if any deviations occurred.	No mention of missing data or dropout rates	Used standardised pain and tenderness evaluations, blood draws. However, it’s unclear if outcome assessors were blinded to group allocation.	The study comprehensively reported on all the measured outcomes, with no indication of selective reporting	While the methodology seems robust in terms of the intervention and measurements used, the lack of explicit information on randomisation, handling of missing data.
Kristoffersen, Christensen, Oliveira (2022) (24) Denmark	?	?	?	?	+	?
Comments	The study employed a blinded randomized trial design. However, specific details on the randomisation method and allocation concealment were not explicitly provided.	Participants were assigned to either a resveratrol or placebo group. The study does not provide detailed information on adherence to the supplementation protocol or if any deviations occurred.	No information was provided regarding how missing data, if any, were addressed.	The study used measures like PPT, VAS, isometric ankle dorsiflexion torque, and EMG. However, the calibration and blinding of assessors for these measurements are not specified.	The study comprehensively reported on all the measured outcomes, with no indication of selective reporting	While the methodology seems robust in terms of the intervention and measurements used, the lack of explicit information on randomisation, handling of missing data.

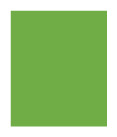
 Low risk of bias

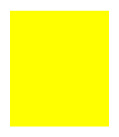
 Unclear risk of bias

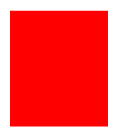
 High risk of bias

## Appendix 1 Database search strategy

### Web of Science (WoS)

The search strategy for Web of Science aims to pinpoint all original articles that investigate the influence of resveratrol supplementation on delayed onset muscle soreness (DOMS) post-physical activity or exercise in adults. The PICO framework (Population, Intervention, Comparison, Outcome) was used to guide the development of this search strategy:

#### Population

The target population encompasses adults (18 years and above) who participated in exercise or physical activity. Keywords such as “Adult*”, “Exercise”, “Physical Activity”, and “Training” were employed to identify this demographic.

#### Intervention

4The focal intervention is the intake of resveratrol. Keywords like “Resveratrol” and “trans-3,5,4’-trihydroxystilbene” was used to pinpoint this intervention.

#### Comparison

The comparison of interest is exercise or physical activity without the intake of resveratrol.

#### Outcome

The primary outcome revolves around the onset and intensity of DOMS, evaluated using biomarkers, standardized tests, or self-report measures. Keywords for this outcome include “Delayed Onset Muscle Soreness”, “DOMS”, “Muscle Pain”, “Muscle Soreness”, “Muscle Ache”, “Biomarkers”, “Biochemical Markers”, “Muscle Damage Markers”, “Standardized Tests”, “Objective Assessment”, “Muscle Function Tests”, “Self-report”, ‘Pain Scale”, “Visual Analog Scale”, “Questionnaire”, “ROM”, and “Range of Motion”.

In the Web of Science Core Collection, the TS=(field) was be employed to search for keywords within the title, abstract, and author keywords:

TS=((“adult*” AND (“exercise” OR “physical activity” OR “training”)) AND (“resveratrol” OR “trans-3,5,4’-trihydroxystilbene”) AND (“delayed onset muscle soreness” OR “DOMS” OR “muscle pain” OR “muscle soreness” OR “biomarkers” OR “biochemical markers” OR “muscle damage markers” OR “standardized tests” OR “objective assessment” OR “muscle function tests” OR “self-report” OR “pain scale” OR “visual analog scale” OR “questionnaire” OR “ROM” OR “Range of Motion”))

### PubMed (Medline)

For the PubMed (Medline) search, a combination of Medical Subject Headings (MeSH) terms, relevant keywords, and synonyms were employed to ensure a comprehensive identification of eligible studies. The same PICO framework as WoS was used to guide the development of this search strategy.

The search strategy encompassed the following MeSH terms and keywords:

((“Adult”[MeSH] OR “Exercise”[Title/ Abstract] OR “Physical Activity”[Title/ Abstract] OR “Training”[Title/Abstract]) AND (“Resveratrol”[MeSH] OR “trans-3,5,4’-trihydroxystilbene”[Title/Abstract]) AND (“Delayed Onset Muscle Soreness”[Title/ Abstract] OR “DOMS”[Title/Abstract] OR “Muscle Pain”[Title/Abstract] OR “Muscle Soreness”[Title/Abstract] OR “Muscle Ache”[Title/Abstract] OR “Biomarkers”[Title/Abstract] OR “Biochemical Markers”[Title/Abstract] OR “Muscle Damage Markers”[Title/Abstract] OR “Standardized Tests”[Title/Abstract] OR “Objective Assessment”[Title/ Abstract] OR “Muscle Function Tests”[Title/Abstract] OR “Self-report”[Title/Abstract] OR “Pain Scale”[Title/Abstract] OR “Visual Analog Scale”[Title/Abstract] OR “Questionnaire”[Title/Abstract] OR “ROM”[Title/Abstract] OR “Range of Motion”[Title/Abstract])).

### Scopus

The search strategy for Scopus was conducted using the Scopus database. The same PICO framework as WoS and Pubmed (Medline) was used to guide the development of this search strategy. The search was not limited by date, language, or publication status to ensure a comprehensive review of the available literature. The search strategy was as follows:

(TITLE-ABS-KEY(“Adult” OR “Exercise” OR “Physical Activity” OR “Training”) AND TITLE-ABS-KEY(“Resveratrol” OR “trans-3,5,4’-trihydroxystilbene”) AND TITLEABS-KEY(“Delayed Onset Muscle Soreness” OR “DOMS” OR “Muscle Pain” OR “Muscle Soreness” OR “Biomarkers” OR “Biochemical Markers” OR “Muscle Damage Markers” OR “Standardized Tests” OR “Objective Assessment” OR “Muscle Function Tests” OR “Self-report” OR “Pain Scale” OR “Visual Analog Scale” OR “Questionnaire”))

### Cochrane Library (CENTRAL)

The search strategy for the Cochrane Library was conducted using the Cochrane Central Register of Controlled Trials (CENTRAL) database. The same PICO framework as WoS, Pubmed (Medline) and Scopus was used to guide the development of this search strategy. The search was not limited by date, language, or publication status to ensure a comprehensive review of the available literature. The search strategy was as follows:

((Adult* OR Exercise OR “Physical Activity” OR Training) AND (Resveratrol OR “trans-3,5,4’-trihydroxystilbene”) AND (“Delayed Onset Muscle Soreness” OR DOMS OR “Muscle Pain” OR “Muscle Soreness” OR Biomarkers OR “Biochemical Markers” OR “Muscle Damage Markers” OR “Standardized Tests” OR “Objective Assessment” OR “Muscle Function Tests” OR Self-report OR “Pain Scale” OR “Visual Analog Scale” OR Questionnaire))

## References

[1] Armstrong RB (1984). Mechanisms of exercise-induced delayed onset muscular soreness: a brief review. Med Sci Sports Exerc.

[2] Mizumura K, Taguchi T (2016). Delayed onset muscle soreness: Involvement of neurotrophic factors. J Physiol Sci.

[3] Mautner K, Sussman WI (2016). Delayed Onset Muscle Soreness. Curr Sports Med Rep.

[4] Meng X, Zhou J, Zhao CN, Gan RY, Li HB (2020). Health benefits and molecular mechanisms of resveratrol: a narrative review. Foods.

[5] Kumar A, PN, Kumar M, Jose A, Tomer V, Oz E (2023). Major phytochemicals: recent advances in health benefits and extraction method. Molecules.

[6] Baltaci SB, Mogulkoc R, Baltaci AK (2016). Resveratrol and exercise. Biomed Rep.

[7] Huang CC, Lee MC, Ho CS, Hsu YJ, Ho CC, Kan NW (2021). Protective and recovery effects of resveratrol supplementation on exercise performance and muscle damage following acute plyometric exercise. Nutrients.

[8] Wang L, Xu Z, Ling D, Li J, Wang Y, Shan T (2022). The regulatory role of dietary factors in skeletal muscle development, regeneration and function. Crit Rev Food Sci Nutr.

[9] Myers MJ, Shepherd DL, Durr AJ, Stanton DS, Mohamed JS, Hollander JM (2019). The role of SIRT1 in skeletal muscle function and repair of older mice. J Cachexia Sarcopenia Muscle.

[10] Bennett BT, Mohamed JS, Alway SE (2013). Effects of resveratrol on the recovery of muscle mass following disuse in the plantaris muscle of aged rats. PLoS ONE.

[11] Rogers R, Baumann C, Otis J (2015). Recovery of skeletal muscle function following injury is not augmented by acute resveratrol supplementation. Int J Clin Exp Physiol.

[12] Hsu YJ, Ho CS, Lee MC, Ho CS, Huang CC, Kan NW (2020). Protective effects of resveratrol supplementation on contusion induced muscle injury. Int J Med Sci.

[13] Meng T, Xiao D, Muhammed A, Deng J, Chen L, He J (2021). Anti-inflammatory action and mechanisms of resveratrol. Molecules.

[14] de la Lastra CA, Villegas I (2007). Resveratrol as an antioxidant and pro-oxidant agent: mechanisms and clinical implications. Biochem Soc Trans.

[15] Wu RE, Huang WC, Liao CC, Chang YK, Kan NW, Huang CC (2013). Resveratrol protects against physical fatigue and improves exercise performance in mice. Molecules.

[16] Tsao JP, Liu CC, Wang HF, Bernard JR, Huang CC, Cheng IS (2021). Oral resveratrol supplementation attenuates exercise-induced interleukin-6 but not oxidative stress after a high intensity cycling challenge in adults. Int J Med Sci.

[17] Laupheimer MW, Perry M, Benton S, Malliaras P, Maffulli N (2014). Resveratrol exerts no effect on inflammatory response and delayed onset muscle soreness after a marathon in male athletes.: A randomised, double-blind, placebo-controlled pilot feasibility study. Transl Med UniSa.

[18] Løkken N, Khawajazada T, Storgaard JH, Raaschou-Pedersen D, Christensen ME, Hornsyld TM (2021). No effect of resveratrol in patients with mitochondrial myopathy: a cross-over randomized controlled trial. J Inherit Metab Dis.

[19] Macedo RCS, Vieira A, Marin DP, Otton R (2015). Effects of chronic resveratrol supplementation in military firefighters undergo a physical fitness test - A placebo-controlled, double blind study. Chem Biol Interact.

[20] Jo E, Bartosh R, Auslander AT, Directo D, Osmond A, Wong MWH (2019). Post-exercise recovery following 30-day supplementation of trans-resveratrol and polyphenol-enriched extracts. Sports.

[21] O’connor PJ, Caravalho AL, Freese EC, Cureton KJ (2013). Grape consumption’s effects on fitness, muscle injury, mood, and perceived health. International Journal of Sport Nutrition and Exercise Metabolism.

[22] Kawamura A, Aoi W, Abe R, Kobayashi Y, Kuwahata M, Higashi A (2021). Astaxanthin-, β-Carotene-, and Resveratrol-Rich Foods Support Resistance Training-Induced Adaptation. Antioxidants.

[23] Udani J, Singh B, Sandoval E (2008). Effect of a combination dietary supplement product (Bounce-Back^™^) on the signs and symptoms of delayed onset muscle soreness after eccentric exercise: a randomized, double-blind, placebo-controlled, crossover pilot study. J Int Soc Sports Nutr.

[24] Kristoffersen SS, Christensen AJ, Oliveira AS (2022). Resveratrol administration reduces pain perception but does not attenuate force loss following exercise-induced muscle damage. Sport Sci Health.

[25] Page MJ, McKenzie JE, Bossuyt PM, Boutron I, Hoffmann TC, Mulrow CD (2021). The PRISMA 2020 statement: an updated guideline for reporting systematic reviews. BMJ.

[26] Sterne JAC, Savović J, Page MJ, Elbers RG, Blencowe NS, Boutron I (2019). RoB 2: a revised tool for assessing risk of bias in randomised trials. BMJ.

[27] Ranchordas MK, Rogerson D, Soltani H, Costello JT (2017). Antioxidants for preventing and reducing muscle soreness after exercise. Cochrane Database of Systematic Reviews.

[28] Dolinsky VW, Jones KE, Sidhu RS, Haykowsky M, Czubryt MP, Gordon T (2012). Improvements in skeletal muscle strength and cardiac function induced by resveratrol during exercise training contribute to enhanced exercise performance in rats. J Physiol.

[29] Lagouge M, Argmann C, Gerhart-Hines Z, Meziane H, Lerin C, Daussin F (2006). Resveratrol improves mitochondrial function and protects against metabolic disease by activating SIRT1 and PGC-1α. Cell.

[30] Ferrucci L, Fabbri E (2018). Inflammageing: chronic inflammation in ageing, cardiovascular disease, and frailty. Nat Rev Cardiol.

[31] Marin DP, Bolin AP, Campoio TR, Guerra BA, Otton R (2013). Oxidative stress and antioxidant status response of handball athletes: implications for sport training monitoring. Int Immunopharmacol.

[32] Henry LA, Witt DM (2002). Resveratrol: phytoestrogen effects on reproductive physiology and behavior in female rats. Horm Behav.

[33] Gehm BD, McAndrews JM, Chien PY, Jameson JL (1997). Resveratrol, a polyphenolic compound found in grapes and wine, is an agonist for the estrogen receptor. Proceedings of the National Academy of Sciences.

[34] Baur JA, Pearson KJ, Price NL, Jamieson HA, Lerin C, Kalra A (2006). Resveratrol improves health and survival of mice on a high-calorie diet. Nature.

[35] Wang XL, Li T, Li JH, Miao SY, Xiao XZ (2017). The Effects of Resveratrol on Inflammation and Oxidative Stress in a Rat Model of Chronic Obstructive Pulmonary Disease. Molecules.

[36] Sun S, Zhang M, Yang Q, Shen Z, Chen J, Yu B (2017). Resveratrol suppresses lipoprotein-associated phospholipase A_2_ expression by reducing oxidative stress in macrophages and animal models. Mol Nutr Food Res.

[37] Toniolo L, Concato M, Giacomello E (2023). Resveratrol, a multitasking molecule that improves skeletal muscle health. Nutrients.

[38] McCalley A, Kaja S, Payne A, Koulen P (2014). Resveratrol and calcium signaling: molecular mechanisms and clinical relevance. Molecules.

[39] Robb EL, Stuart JA (2014). The stilbenes resveratrol, pterostilbene and piceid affect growth and stress resistance in mammalian cells via a mechanism requiring estrogen receptor beta and the induction of Mn-superoxide dismutase. Phytochemistry.

[40] Robb EL, Page MM, Wiens BE, Stuart JA (2008). Molecular mechanisms of oxidative stress resistance induced by resveratrol: specific and progressive induction of MnSOD. Biochem Biophys Res Commun.

[41] Ren Z, Wang L, Cui J, Huoc Z, Xue J, Cui H (2013). Resveratrol inhibits NF-kB signaling through suppression of p65 and IkappaB kinase activities. Pharmazie.

[42] Vanamala J, Reddivari L, Radhakrishnan S, Tarver C (2010). Resveratrol suppresses IGF-1 induced human colon cancer cell proliferation and elevates apoptosis via suppression of IGF-1R/Wnt and activation of p53 signaling pathways. BMC Cancer.

[43] de Sá Coutinho D, Pacheco M, Frozza R, Bernardi A (2018). Anti-inflammatory effects of resveratrol: mechanistic insights. Int J Mol Sci.

[44] Kan NW, Lee MC, Tung YT, Chiu CC, Huang CC, Huang WC (2018). The synergistic effects of resveratrol combined with resistant training on exercise performance and physiological adaption. Nutrients.

[45] Skroza D, Generalić Mekinić I, Svilović S, Šimat V, Katalinić V (2015). Investigation of the potential synergistic effect of resveratrol with other phenolic compounds: a case of binary phenolic mixtures. Journal of Food Composition and Analysis.

[46] Wei Y, Yang S, Zhang L, Dai L, Tai K, Liu J (2020). Fabrication, characterization and in vitro digestion of food grade complex nanoparticles for co-delivery of resveratrol and coenzyme Q10. Food Hydrocoll.

[47] XU WANG N, LI C, WANG N, WANG F, LI FZ (2022). Synergistic anti-inflammatory effects of resveratrol and vitamin E in lipopolysaccharide-induced RAW264.7 cells. Food Science and Technology.

[48] Di Santo A, Mezzetti A, Napoleone E, Di Tommaso R, Donati MB, De Gaetano G (2003). Resveratrol and quercetin down-regulate tissue factor expression by human stimulated vascular cells. Journal of Thrombosis and Haemostasis.

[49] Park HJ, Yang JY, Ambati S, Della-Fera MA, Hausman DB, Rayalam S (2008). Combined effects of genistein, quercetin, and resveratrol in human and 3T3-L1 adipocytes. J Med Food.

[50] Hsu CN, Lin YJ, Tain YL (2019). Maternal exposure to bisphenol a combined with high-fat diet-induced programmed hypertension in adult male rat offspring: Effects of resveratrol. Int J Mol Sci.

[51] Cheng J, Xia X, Rui Y, Zhang Z, Qin L, Han S (2016). The combination of 1α,25dihydroxyvitaminD3 with resveratrol improves neuronal degeneration by regulating endoplasmic reticulum stress, insulin signaling and inhibiting tau hyperphosphorylation in SH-SY5Y cells. Food and Chemical Toxicology.

[52] Salehi B, Mishra A, Nigam M, Sener B, Kilic M, Sharifi-Rad M (2018). Resveratrol: a double-edged sword in health benefits. Biomedicines.

[53] Shaito A, Posadino AM, Younes N, Hasan H, Halabi S, Alhababi D (2020). Potential adverse effects of resveratrol: a literature review. Int J Mol Sci.

